# Role of the Bronchoalveolar Lavage in Noncritically Ill Patients during the SARS-CoV-2 Epidemic

**DOI:** 10.1155/2020/9012187

**Published:** 2020-12-17

**Authors:** Olivier Taton, Emmanuelle Papleux, Benjamin Bondue, Christiane Knoop, Sébastien Van Laethem, Alain Bauler, Delphine Martiny, Isabel Montesinos, Marie-Luce Delforge, Kahina Elmaouhab, Dimitri Leduc

**Affiliations:** ^1^Department of Pneumology, Hôpital Erasme, Université Libre de Bruxelles, Brussels, Belgium; ^2^Department of Pneumology, Hôpitaux Iris Sud, Brussels, Belgium; ^3^Department of Microbiology, Laboratoire Hospitalier Universitaire de Bruxelles-Universitair Laboratorium Brussel (LHUB-ULB), Brussels, Belgium; ^4^Department of Virology, Hôpital Erasme, Université Libre de Bruxelles, Brussels, Belgium; ^5^Department of Infectious Diseases, Hôpital Erasme, Université Libre de Bruxelles, Brussels, Belgium

## Abstract

**Background:**

Bronchoalveolar lavage (BAL) is currently not recommended in noncritically ill patients for the diagnosis of SARS-CoV-2 infection. Indeed, the diagnosis is based on the RT-PCR test on a nasopharyngeal swab (NPS) and abnormal findings on the chest CT scan. However, the sensitivity of the NPS and the specificity of the chest CT scan are low. Results of BAL in case of negative NPS testing are underreported, especially in the subgroup of immunocompromised patients.

**Objectives:**

The added value of BAL in the management of unstable, but noncritically ill patients, suspected of having SARS-CoV-2 infection despite one previous negative NPS and the side effects of the procedure for the patients and the health-care providers, were assessed during the epidemic peak of the COVID-19 outbreak in Belgium.

**Methods:**

This multicentric study included all consecutive noncritically ill patients hospitalized with a clinical and radiological suspicion of SARS-CoV-2 infection but with a negative NPS. BAL was performed according to a predefined decisional algorithm based on their state of immunocompetence, the chest CT scan features, and their respiratory status.

**Results:**

Among the 55 patients included in the study, 14 patients were diagnosed with a SARS-CoV-2 infection. Interestingly, there was a relationship between the cycle threshold of the RT-PCR and the interval of time between the symptom onset and the BAL procedure (Pearson′s correlation coefficient = 0.8, *p* = 0.0004). Therapeutic management was changed in 33 patients because another infectious agent was identified in 23 patients or because an alternative diagnosis was made in 10 patients. In immunocompromised patients, the impact of BAL was even more marked (change in therapy for 13/17 patients). No significant adverse event was noted for patients or health-care staff. All health-care workers remained negative for SARS-CoV-2 NPS and serology at the end of the study.

**Conclusions:**

In this real-life study, BAL can be performed safely in selected noncritically ill patients suspected of SARS-CoV-2 infection, providing significant clinical benefits that outweigh the risks.

## 1. Introduction

A novel coronavirus outbreak (severe acute respiratory syndrome coronavirus 2 (SARS-CoV-2)) is spreading all over the world [1]. The diagnosis of this new virus infection is based on the identification of viral RNA on nasopharyngeal swabs and typical abnormal findings on chest CT scan [2].

To date, bronchoalveolar lavage (BAL), which is a cornerstone exam in the setting of respiratory infections [3], is not recommended in noncritically ill patients because the virus is highly contagious and the procedure could increase the risk of transmission to health-care providers [4]. If a patient is highly suspected for SARS-CoV-2 infection despite a negative nasopharyngeal swab, it is recommended to repeat the nasopharyngeal swab [5].

There are, however, several drawbacks to this recommendation. First, the sensitivity of the nasopharyngeal swab and the specificity of the chest CT scan for the diagnosis of SARS-CoV-2 infection are low [6, 7]. A number of external factors may affect the viral nucleic acid detection including the adequacy of the nasopharyngeal swab as well as the timing of sampling related to the disease stage (i.e., higher viral loads early in the course of the infection) [8, 9]. Second, the turn-around time for reverse transcriptase polymerase chain reaction (RT-PCR) results was initially quite long (several days), potentially leaving patients without a diagnosis. Finally, this paradigm may not apply to immunocompromised patients, who are at higher risk of developing opportunistic infections [10], which could present with the same radiological expression as SARS-CoV-2 infection. Indeed, there could be overlap of CT imaging features between SARS-CoV-2 and other pulmonary potentially treatable diseases [9].

By establishing a definite or alternative diagnosis, we hypothesize that the expected benefits of BAL could outweigh the potential side effects of the bronchoscopic procedure and the risks for the health-care team in a subset of noncritically ill patients.

The main purpose of our study was therefore to assess the added value of BAL in the management of unstable, but noncritically ill patients, suspected of having SARS-CoV-2 infection despite at least one previous negative nasopharyngeal swab during the epidemic peak in Belgium [11]. We developed a decisional algorithm, allowing an *a priori* selection of patients eligible for the BAL procedure. We also aimed to evaluate the additional information provided by BAL fluid analysis, as well as the side effects of the procedure for the patients and the health-care providers.

## 2. Materials and Methods

### 2.1. Study Design

This retrospective study was conducted at Erasme University Hospital and Iris Sud Hospital in Brussels, Belgium, from March, 13th, 2020 to April, 30th, 2020. This period corresponds to the epidemic peak of the SARS-CoV-2 infection in Belgium [11]. The study protocol was approved by the ethics committees of both participating hospitals (Ref. Nr.: P2020/230) with a waiver of informed consent.

### 2.2. Population

Consecutive patients were considered for inclusion in the study if they fulfilled the following criteria: age over 18 years and clinical features requiring hospital admission for suspected SARS-CoV-2 infection with a first negative nasopharyngeal swab and a chest CT scan performed within the last 48 hours. Patients requiring intensive care unit (ICU) admission were excluded.

Suspicion of SARS-CoV-2 infection was defined as typical clinical presentation (more than one of the following signs or symptoms: fever, cough, dyspnea, hypoxemia, and flu-like syndrome) associated with any abnormal findings on chest CT scan [12].

Typical chest CT scan features of a SARS-CoV-2 infection included the presence of peripheral bilateral ground glass opacities with or without consolidations and intralobular septa thickening. Atypical chest CT scan manifestations, including bronchial wall thickening, pleural effusions, lymphadenopathy, and pulmonary nodules, were suggestive of another more likely diagnosis than SARS-CoV-2 infection [12].

**Figure 1:**
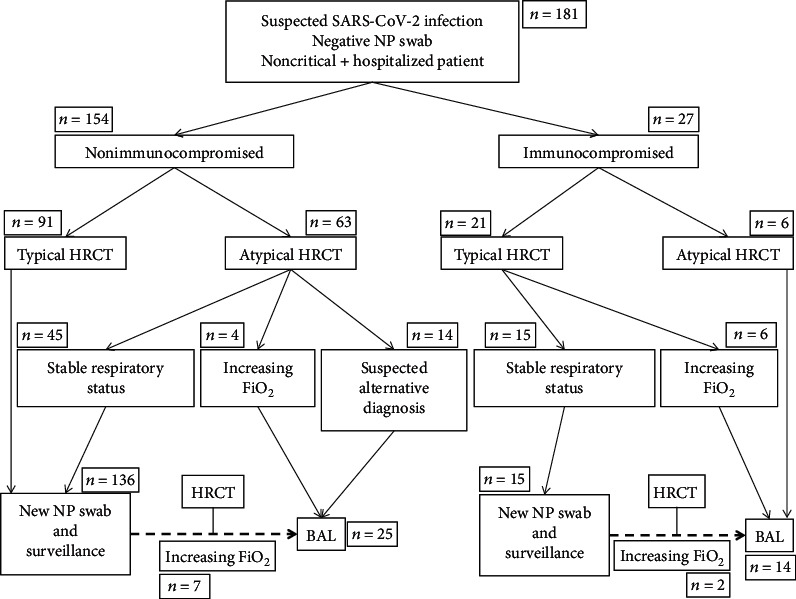
Decisional algorithm followed for indication of bronchoalveolar lavage (BAL) in noncritically ill patients with suspicion of SARS-CoV-2 infection, with a first negative nasopharyngeal (NP) swab. Stable respiratory status means that the patient has no increase of oxygen need in the past 48 hours. Abbreviations: NP—nasopharyngeal; BAL—bronchoalveolar lavage; HRCT—high-resolution chest CT scan.

BAL was performed in patients who were suspected to have SARS-CoV-2 infection but who had at least one previous negative nasopharyngeal swab, according to a predefined decisional algorithm (Figure 1), which took into account the patient's state of immunocompetence/immunosuppression, the typical or atypical presentation on chest CT scan, and the clinical stability/instability of the patients with regard to supplemental oxygen therapy. So, BAL procedures (rather than repetitive nasopharyngeal swabs) were performed in patients requiring increased oxygen need, suggesting worsening of the respiratory function; patients with atypical CT scan suggestive of an alternate diagnosis than SARS-CoV-2 infection; and immunocompromised patients with atypical CT scan. Clinical outcomes were followed for at least 4 weeks after the BAL procedure.

### 2.3. Endoscopic Interventions

BAL was performed under local anesthesia with lidocaine 1% [13]. The exams were performed by experienced bronchoscopists, at bedside in a SARS-CoV-2 isolation room, using a disposable video-bronchoscope (Ambu® aScope™, Ballerup, Denmark). Airborne precautions, including FFP2 mask and personal protective equipment, were used. BAL was performed according to standard procedures [14]. Briefly, at least 100 ml and a maximum of 150 ml of sterile isotonic saline, divided in 50 ml syringes, were instilled through the working channel of the bronchoscope into the most affected segment of the lung. The aliquot aspirated after the first syringe injected was always sent for cytological examination. The subsequent aspirated aliquots were put into sterile containers for further laboratory testing.

### 2.4. Laboratory Testing

Microbiological analysis included microscopic examination; standardized fungal, viral, and bacterial cultures; acid-fast stain; and *Mycobacterium tuberculosis* PCR and culture. The positive cut-off for bacterial culture was 10,000 colony-forming units per milliliter [15].

For SARS-CoV-2 detection, viral RNA extraction was performed by the m2000 mSample Preparation SystemDNA Kit (Abbott, Chicago, IL, USA) using 1000 *μ*l of a manually lysed sample (obtained from a 700 *μ*l sample + 800 *μ*l lysis buffer from a kit) eluted in 90 *μ*l of elution buffer. A quantitative (q)RT-PCR internal control was added at each extraction. qRT-PCR was performed using 10 *μ*l of the extracted sample in the RealStar® SARS-CoV-2 RT-PCR Kit (Altona-Diagnostics, Hamburg, Germany) with a cut-off set at a cycle threshold (Ct) value of 40. Since quantitative results were not available, the Ct was used as a relative measurement of the target concentration in the PCR reaction—the Ct value being inversely correlated with the amount of RNA present. A Ct value of less than 40 was defined as a positive test result indicating a significant viral load in the specimen [8]. A customized TaqMan array card (TAC) was also used for the detection of a larger panel of respiratory pathogens, as described previously [16].

*Aspergillus* galactomannan assay was performed using the one-stage commercialized immunoenzymatic sandwich microplate assay (Platelia *Aspergillus* Ag; Bio-Rad, Temse, Belgium) according to the manufacturer's instructions.

### 2.5. Endpoints of the Study

The primary endpoint of the study was the impact of BAL results on patient management and outcome. Change of therapeutic management is defined as the initiation or a change in antimicrobial therapy, the start of steroid treatment, and/or the transfer of a patient out of the SARS-CoV-2 isolation ward.

Secondary endpoints included the yield of BAL, defined as the rate of positive detection of SARS-CoV-2 infection and/or the identification of another pathogen that could explain the patient's clinical features and evolution; the proportion of coinfections, specifically in the subgroup of immunocompromised patients; and the occurrence of adverse events in patients and health-care staff following the endoscopic procedure. Each health-care staff had a nasopharyngeal swab and a blood sample with SARS-CoV-2 immunoglobulin G (IgG) serology within 4 weeks after inclusion of the last patient of our study.

### 2.6. Statistical Methods

Continuous variables are presented as means with standard deviation (SD) or median with interquartile range (IQR) depending on their distribution and compared with the Mann–Whitney *U* test. Categorical variables are expressed as number (%) and compared by the chi-square test or the Fisher exact test. The correlation between the SARS-CoV-2 viral load expressed as the Ct value obtained by RT-PCR and the interval of time between the day of symptom onset and the day of the BAL procedure was determined by the Pearson correlation test.

A *p* value of less than 0.05 was considered statistically significant. The yield of BAL was characterized by the values of sensitivity and accuracy.

## 3. Results

### 3.1. Patients' Characteristics

Between March, 13th, 2020 and April, 30th, 2020, 261 consecutive noncritically ill patients with clinical and radiological features of a SARS-CoV-2 infection, but with a first negative nasopharyngeal swab, were hospitalized in dedicated isolation wards.

BAL was performed in 55 patients (33 men/22 women, mean age 62 ± 16 years), 35 in Erasme Hospital (center 1) and 20 in Iris Sud Hospital (center 2) (Table 1). No statistically significant differences was noted between both centers regarding demographic and baseline patients' characteristics except for an older age (70 ± 13 vs. 58 ± 16 years, *p* = 0.006) and a higher number of patients under antibiotic treatment at the time of the procedure (9 patients vs. 5 patients, *p* = 0.022) in center 2 (Table 1). Twenty-four patients (44%) had 2 negative nasopharyngeal swabs before the BAL.

Initial typical symptoms of SARS-CoV-2 infection including fever, cough, and dyspnea were observed in 31 patients (56%). According to the initial chest CT scan, 29 patients (53%) showed bilateral, peripheral, and subpleural ground glass opacities while the remaining 26 patients (47%) presented atypical imaging features.

### 3.2. BAL Fluid Analysis

#### 3.2.1. SARS-CoV-2 Infection

Fourteen out of 55 patients (25%) with at least 1 initial negative nasopharyngeal swab and who underwent BAL had a positive SARS-CoV-2 RT-PCR at the end of the workup. Thirteen patients (13/14, 93%) were diagnosed through BAL fluid analysis and 1 patient (1/14, 7%) was diagnosed through a further nasopharyngeal swab performed 2 days after the BAL procedure. Using any of these positive RT-PCR results as reference standard, sensitivity and yield of BAL for SARS-CoV-2 diagnosis were 93% (13/14) and 84% (46/55), respectively.

Of the 14 specimens positive for SARS-CoV-2, only 1 patient (7%) was also positive for an additional respiratory pathogen (*Serratia marcescens*) (Table 2).

**Figure 2:**
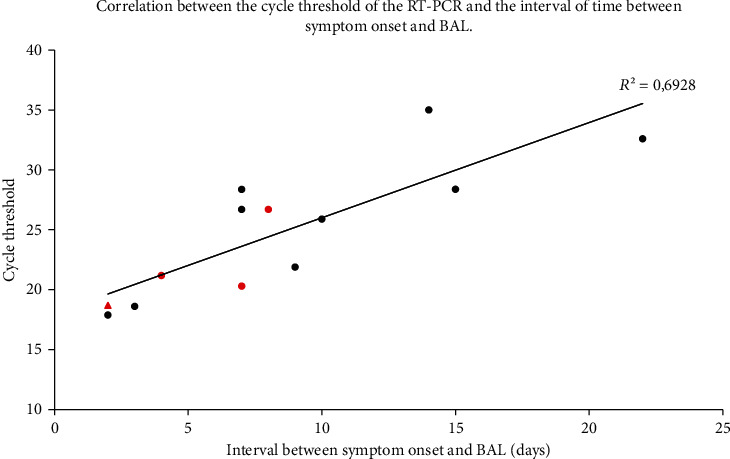
Cycle threshold of the RT-PCR for SARS-CoV-2 in BAL fluid by the interval of time from onset of symptoms at time of BAL procedure in 13 SARS-CoV-2-positive patients. The Pearson correlation coefficient was 0.8, *p* = 0.0004. Dots and triangle represent the patients alive and dead, respectively, 4 weeks after the BAL. Red markers represent the patients admitted to the ICU. Abbreviations: BAL—bronchoalveolar lavage; RT-PCR—reverse transcriptase polymerase chain reaction.

The average time from initial disease onset to the first nasopharyngeal swab was 5.3 ± 5.1 days and to the BAL procedure was 9.1 ± 6.9 days (Table 1).  Figure 2 shows the relationship between the viral load, assessed by the Ct of the RT-PCR on BAL obtained from 13 patients infected with SARS-CoV-2, and the time interval between symptom onset and the BAL procedure. A positive correlation (Pearson′s correlation coefficient = 0.8, *p* = 0.0004) was observed, suggesting that higher viral loads (inversely related to the Ct value) were detected earlier after the symptom onset in specimens obtained from the lower respiratory tract.

#### 3.2.2. Non-SARS-CoV-2 Infection

Twenty-three out of the 55 patients (42%) were diagnosed with a non-SARS-CoV-2 infection using BAL fluid analysis. Specific infectious agents identified in these patients are summarized in Table 2.

In 18 out of the 55 patients (33%), no specific pathogen was detected in BAL fluid either by RT-PCR or by culture. Among these 18 patients, there was a more likely alternative diagnosis in 10 patients (18%). Indeed, 4 patients presented with a cardiogenic pulmonary edema, 2 with a cryptogenic organizing pneumonia exacerbation, 1 with a rheumatoid arthritis-associated interstitial lung disease exacerbation, 1 with a hepatopulmonary syndrome, 1 with hypersensitivity pneumonitis, and 1 with sarcoidosis exacerbation. Each patient had at least one additional negative nasopharyngeal swab for SARS-CoV-2 after the BAL procedure. All these patients received specific treatment with good outcomes.

The final diagnosis remained undetermined in the 8 remaining other patients. In 7 (88%) of them, BAL was performed while they received antibiotics. All of them improved under antibiotic treatment and could return home at the end of antibiotic treatment. We assumed that those patients had bacterial pneumonia based on consistent clinical history and good outcome under antibiotic treatment but, indeed, the BAL results had no impact on their therapeutic management.

### 3.3. Comparison between Immunocompromised and Immunocompetent Patients

The characteristics of immunocompromised patients are summarized in Table 3. No significant difference was observed between the immunocompetent and the immunocompromised group, especially regarding disease severity evaluated by the FiO_2_ (Tables 1 and 3), typical or atypical patterns on chest CT, mortality, ICU admission, and discharge rates (data not shown).

Among the 17 immunocompromised patients, only 2 patients (12%) were diagnosed positive for SARS-CoV-2 compared to 32% (12/38) among immunocompetent patients (Table 3). All patients with a negative BAL for SARS-CoV-2 infection (15 patients, 88%) had at least one further negative nasopharyngeal swab for SARS-CoV-2 after the BAL procedure.

### 3.4. Impact of BAL Results on Patient Management and Outcome

BAL results changed the therapeutic management in 33 patients (60%) because another infectious agent was identified in 23 patients (42%) or because an alternative diagnosis was provided in 10 patients (18%). Among the patients in whom a specific infectious agent was found, the change of therapeutic management consisted in the administration of an antimicrobial treatment and a transfer out of the isolation ward. All patients with an alternative final diagnosis received a specific treatment with a good outcome.

Even more importantly, among the immunocompromised patients, BAL results changed the therapeutic management in 13 patients (76%) because another pathogen was identified in 8 patients (47%) and an alternative diagnosis was made in 5 patients (29%). In the 4 remaining patients, 2 of them were diagnosed with SARS-CoV-2 infection by BAL fluid analysis followed by a slow and progressive improvement of their clinical status and 2 patients with an undetermined diagnosis had good clinical outcome.

### 3.5. Adverse Events

Transient increasing of FiO_2_ (up to a maximum of 60% with no need of ICU admission or invasive ventilation) was the only adverse event related to the BAL procedure in our population. No health-care provider involved in the BAL procedures (8 physicians and 10 nurses) reported any signs or symptoms suggestive of a potential SARS-CoV-2 infection within 4 weeks after inclusion of the last patient of our study, and none tested positive for SARS-CoV-2 on nasopharyngeal swab or for anti-SARS-CoV-2 IgG serology.

## 4. Discussion

Several scientific pulmonology societies have issued a general recommendation against the use of bronchoscopy in nonintubated SARS-CoV-2-suspected patients [4]. In our pulmonology department, a well-equipped bronchoscopy suite is run by several pulmonologists specialized in interventional bronchoscopy. From the outset of the SARS-CoV-2 pandemic, we considered that bronchoscopy with BAL could be of added value in the subset of noncritically ill patients suspected of SARS-CoV-2 infection, but who had at least 1 negative nasopharyngeal swab, and selected for BAL procedure because of (1) unstability from a respiratory point of view (increasing FiO_2_), (2) atypical CT scan suggestive of an alternative diagnosis, or (3) immunodepression and atypical CT scan. We devised an *a priori* decisional algorithm and hypothesized that, in this subgroup of patients, the benefit of bronchoscopy with BAL would outweigh the side effects for the patients and the risks for the health-care team.

We applied our algorithm to all consecutive noncritically ill patients suspected of SARS-CoV-2 infection, but with at least 1 negative nasopharyngeal swab, admitted to our dedicated isolation wards and selected 55 patients, who then underwent BAL. BAL allowed us to establish a final diagnosis of SARS-CoV-2 in 13/55 patients (24%), who previously had had 1 or 2 negative nasopharyngeal swabs. The BAL fluid analysis also identified pathogens other than SARS-CoV-2 in 23 patients (42%), and finally, a negative BAL strengthened an alternate diagnosis in 10 other patients (18%). In summary, in this carefully selected subset of patients, BAL results gave useful information in 46 patients (84%), and a significant change in therapeutic management was possible in 33 patients (60%) either by starting or adapting the antimicrobial treatment (*n* = 23) or by giving a specific treatment when an alternative diagnosis was considered (*n* = 10).

The impact of the BAL fluid analysis on patient management was even more marked in the subgroup of immunocompromised patients. In 13 of 17 patients (76%), the results of BAL fluid analysis led to a modification of therapy (change in antimicrobial treatment (*n* = 8) or specific treatment in case of an alternative diagnosis (*n* = 5)).

Only minor adverse events were observed in patients (transient increasing of FiO_2_) and, by taking the maximum care in the protection used, no health-care staff reported any symptoms of SARS-CoV-2 infection. Each staff member had a negative nasopharyngeal swab for SARS-CoV-2 and a negative IgG serology at the end of the study. This could suggest that performing BAL with adequate protection equipment in nonintubated patient is safe.

The reference standard for the diagnosis of SARS-CoV-2 infection is RT-PCR applied to respiratory tract specimens [17]. In a recent study from China including 205 patients, it was reported that BAL fluid specimens showed the highest positivity rate for the diagnosis of SARS-CoV-2 compared to specimens from other sites of the respiratory tract (93% (14/15) compared with only 32% (126/398) for pharyngeal swabs) [18]. However, in this series, BAL was performed in only 15 patients, which corresponded to 7% of the population. Here, we report on a larger series as we performed 55 BAL procedures, which corresponded to more than 20% of our eligible population. In our series, we have observed a similar sensitivity of BAL (13/14, 93%) for the diagnosis of SARS-CoV-2. Our results are also in accordance with previous studies performed during the SARS-associated coronavirus outbreak in 2003 [19] and during the current SARS-CoV-2 epidemic [17], showing that the positivity rate of RT-PCR was greater in lower respiratory tract specimens compared to upper respiratory tract samples.

Interestingly, and for the first time on BAL samples, we have shown a correlation between the viral load and the time of sampling from symptom onset. The Ct values gradually increased with time interval from symptom onset suggesting that the viral loads in BAL fluid gradually decreased over time with less potential for transmissibility.

Patients with SARS-CoV-2 disease were found to be infrequently coinfected with other respiratory pathogens as shown in a large series of 99 cases from China [20]. Our results are in line with this previous study as the rate of coinfection in SARS-CoV-2-positive noncritically ill patients was 7% (1/14), significantly lower than in SARS-CoV-2-negative patients (23/41, 56%, *p* = 0.002).

In our study, the diagnosis remained undetermined after the BAL procedure in 8 patients. We assumed that these patients could have bacterial pneumonia, with no specific pathogen identified in BAL fluid, but with a favorable clinical and radiological outcome under antimicrobial therapy. In a study including 35 heart transplant recipients [21], the diagnostic accuracy of bronchoscopic samples in bacterial pneumonia was low, most likely due to empiric antibiotic therapy that was used widely before bronchoscopy, as was the case in 7 out of our 8 patients.

Some limitations of this study should be acknowledged. First, the study included consecutive patients but results were analyzed retrospectively. The sample size was relatively small even after the involvement of 2 centers. This is probably due to the strict indications for BAL procedures following a decisional algorithm, during a limited period of time. However, currently, only case reports have been published on the usefulness of BAL fluid analysis after negative nasopharyngeal swab for SARS-CoV-2 [22–25]. A second limitation is that RT-PCR results for SARS-CoV-2 on BAL samples were used as reference to calculate the sensitivity and accuracy of the test. Finally, our patients were highly selected with mild-to-moderate disease, preventing generalization of our results.

In conclusion, our data and analysis have shown in a real-life study during the SARS-CoV-2 pandemic that BAL samples, obtained through bronchoscopy, can be performed in noncritically ill patients suspected of SARS-CoV-2 infection, with clinical benefits that outweigh the risks with the condition of properly selecting the patients. We suggest to nuance international recommendations that BAL may be useful in the setting of suspected SARS-CoV-2 infection in selected patients. Further studies on a larger series of patients are necessary to validate the proposed decisional algorithm aimed at selecting the patients who will benefit the most.

## Figures and Tables

**Table 1:** Demographic and baseline characteristics. Typical symptoms of SARS-CoV-2 infection correspond to the presence of more than one of the following signs or symptoms: fever, cough, dyspnea, hypoxemia, or flu-like syndrome. Typical chest CT scan features of a SARS-CoV-2 infection include the presence of peripheral bilateral ground glass opacities with or without consolidations and intralobular septa thickening.

	All (*n* = 55)	Center 1 (*n* = 35)	Center 2 (*n* = 20)	*p* value^∗^
Age (y)	62.5 ± 15.8	58.1 ± 15.9	70.1 ± 12.7	0.006
Male *n* (%)	33 (60%)	20 (57%)	13 (65%)	0.775
Comorbidities *n* (%)				
Chronic pulmonary diseases	14 (25%)	8 (23%)	6 (30%)	0.751
Cardiovascular diseases	26 (47%)	18 (51%)	8 (40%)	0.575
Diabetes	8 (14%)	5 (14%)	3 (15%)	>0.999
Digestive diseases	8 (14%)	6 (17%)	2 (10%)	0.696
Kidney diseases	8 (14%)	6 (17%)	2 (10%)	0.696
Immunocompromised *n* (%)	17 (31%)	14 (40%)	3 (15%)	0.072
Typical symptoms of SARS-CoV-2 infection *n* (%)	31 (56%)	19 (54%)	12 (60%)	0.781
Typical SARS-CoV-2 chest CT features *n* (%)	29 (53%)	17 (49%)	12 (60%)	0.575
Number of patients with 2 negative NP swab before BAL *n* (%)	24 (44%)	16 (46%)	8 (40%)	0.781
Time between the symptom onset and the first NP swab (days ± SD)	5.3 ± 5.1	5.5 ± 5.5	5.1 ± 4.9	0.789
Time between the symptom onset and the BAL (days ± SD)	9.1 ± 6.9	9.1 ± 6.7	9.1 ± 7.3	>0.999
FiO_2_ (%, mean ± SD)				
All	29.4 ± 10.6	30.4 ± 11.5	27.6 ± 8.7	0.349
Immunocompromised	30.8 ± 14.7	31.6 ± 16.1	27 ± 3	0.637
Nonimmunocompromised	28.7 ± 8.4	30 ± 7.4	27.4 ± 9.3	0.183
Treatment at the time of the BAL *n* (%)				
Antibiotics	14 (25%)	5 (14%)	9 (45%)	0.022
Hydroxychloroquine	17 (31%)	10 (29%)	7 (35%)	0.767
Antiviral	0	0	0	NA

^∗^ *p* value: difference between center 1 and center 2. Abbreviations: NP—nasopharyngeal; SD—standard deviation; y—years; BAL—bronchoalveolar lavage; NA—not applicable.

**Table 2:** Bronchoalveolar lavage results.

	SARS-CoV-2 positive (*n* = 14)	SARS-CoV-2 negative (*n* = 41)
Pathogens		
SARS-CoV-2	13 (93%)	0
Other pathogens	1 (7%)	23 (56%)^∗^
*Bacteria*	1 (7%)	14 (34%)
*Streptococcus pneumoniae*	0	2 (5%)
*Streptococcus constellatus*	0	1 (2%)
*Haemophilus pneumoniae*	0	3 (7%)
*Serratia marcescens*	1 (7%)	0
*Escherichia coli*	0	2 (5%)
*Acinetobacter baumanii*	0	1 (2%)
*Prevotella* sp.	0	1 (2%)
*Chlamydia pneumoniae*	0	2 (5%)
*Mycoplasma pneumoniae*	0	1 (2%)
*Pneumocystis jirovecii*	0	1 (2%)
*Mycobacterium tuberculosis*	0	1 (2%)
*Virus*	0	7 (17%)
Influenza A	0	4 (10%)
Metapneumovirus	0	1 (2%)
Adenovirus	0	1 (2%)
Herpes simplex virus		1 (2%)
*Fungi*	0	1 (2%)
*Aspergillus fumigatus*	0	1 (2%)^†^
No pathogen	0	18 (44%)
Outcome at 4 weeks after BAL		
Death	1 (7%)	4 (10%)
ICU	3 (21%)^∗^	0
6 (43%)	0	SARS-CoV-2-dedicated unit hospitalisation
1 (7%)	5 (12%)	Ward hospitalisation
Discharged	3 (21%)	32 (78%)^∗^

^∗^ *p* < 0.05 for comparisons between SARS-CoV-2-positive and negative patients. ^†^Galactomannan value: 6. Abbreviations: BAL—bronchoalveolar lavage; ICU—intensive care unit.

**Table 3:** Characteristics of immunocompromised patients.

Patients	Age (y)	Gender	Origin of immunodepression	Other comorbidities	FiO_2_ at the time of BAL	Micorbiological results	Final diagnosis
1	53	M	Glucocorticoids	RA-ILD	28	—	RA-ILD exacerbation
2	61	F	Liver transplant	CKI, HT	21	*Haemophilus influenzae*	*Haemophilus influenzae* pneumonia
3	63	M	Kidney transplant	CKI, HT, cardiac failure	30	*Pneumocystis jirovecii*	*Pneumocystis jirovecii* infection
4	20	M	Heart transplant	Cardiac failure	21	Influenza A	Influenza A infection
5	35	M	Glucocorticoids	Ulcerative colitis	21	—	COP
6	53	F	Glucocorticoids	Acute alcoholic hepatitis, liver failure	80	Influenza A	Influenza A infection
7	68	F	Glucocorticoids	Sarcoidosis, HT, diabetes	35	SARS-CoV-2	SARS-CoV-2 infection
8	71	M	Kidney transplant	HT, multiple myeloma	24	Metapneumovirus	Metapneumovirus infection
9	48	M	Untreated HIV	HT	27	—	Undetermined
10	81	M	Chemotherapy	CML, CKI	30	—	Undetermined
11	41	F	Combination of immunosuppresive agents	Ankylosing spondylitis	25	*Chlamydia pneumoniae*	*Chlamydia pneumoniae* infection
12	74	F	Heart transplant	CKI, HT, diabetes	21	SARS-CoV-2	SARS-CoV-2 infection
13	58	F	Glucocorticoids	Severe asthma	24	*Aspergillus fumigatus*	Invasive aspergillosis
14	38	F	Glucocorticoids	Acute alcoholic hepatitis, liver failure	21	—	Hepatopulmonary syndrome
15	71	M	Chemotherapy	CLL, CKI	30	Herpes simplex virus	Herpes simplex virus infection
16	71	M	Liver transplant	HT, AF	35	—	Cardiogenic pulmonary edema
17	64	M	Sarcoidosis	Emphysema	50	—	Sarcoidosis exacerbation

Abbreviations: M—male; F—female; HIV—human immunodeficiency virus; RA-ILD—rheumatoid arthritis-associated interstitial lung disease; CKI—chronic kidney injury; HT—hypertension; CML—chronic myeloid leukemia; COP—cryptogenic organizing pneumonia; CLL—chronic lymphoid leukemia; AF—atrial fibrillation.

## Data Availability

All the data are available on request from the authors.

## Abbreviations

BAL: Bronchoalveolar lavage
Ct: Cycle threshold
ICU: Intensive care unit
IgG: Immunoglobulin G
IQR: Interquartile range
RT-PCR: Reverse transcriptase polymerase chain reaction
qRT-PCR: Quantitative reverse transcriptase polymerase chain reaction
SD: Standard deviation
TAC: TaqMan Array card.
